# Exploring the comorbidity of musculoskeletal and personality disorders among adults: a scoping review protocol

**DOI:** 10.1186/s13643-021-01721-6

**Published:** 2021-06-21

**Authors:** Shae E. Quirk, Heli Koivumaa-Honkanen, Risto Honkanen, Jeremi Heikkinen, Bianca E. Kavanagh, Lana J. Williams

**Affiliations:** 1 Deakin University, Institute for Mental and Physical Health and Clinical Translation, School of Medicine, Geelong, Victoria Australia; 2 Institute of Clinical Medicine, Psychiatry, University of Eastern Finland, Kuopio, Finland; 3 Institute of Clinical Medicine, Kuopio Musculoskeletal Research Unit (KMRU), University of Eastern Finland, Kuopio, Finland; 4 Mental Health and Wellbeing Center, Kuopio University Hospital, Kuopio, Finland; 5 University Hospital Geelong, Barwon Health, Geelong, Victoria Australia

**Keywords:** Comorbidity, Mental health, Musculoskeletal disorders, Musculoskeletal diseases, Personality disorder, Psychiatry

## Abstract

**Background:**

Separately, mental and musculoskeletal disorders (MSDs) are prevalent across the life course and are leading contributors to disability worldwide. While people with personality disorder (PD) have been shown to have an increased risk of certain physical health comorbidities—associations with MSDs have not been thoroughly explored. The proposed scoping review aims to explore the existing clinical- and population-based literature on the comorbidity of PD and MSDs among adults ≥ 18 years and the burden associated with their comorbidity, identify knowledge gaps on this topic, and propose recommendations for future research.

**Methods:**

This protocol describes the methodology to undertake the scoping review. It is guided by Arksey and O’Malley’s framework and the extensions recommended by the Joanna Briggs Institute. A comprehensive search strategy will be used to identify relevant articles, which will be underpinned by Population, Concept, and Context (PCC) inclusion criteria. One author will perform the search and two authors will independently screen titles/abstracts followed by a full-text review for articles considered relevant. The supervising author will confirm the final selection of articles to be included. One author will extract relevant information from the articles using a predetermined charting form, while a second will perform validation of all information entered.

**Discussion:**

Information will be synthesised to inform a discussion of what is known regarding associations between PD and MSDs, and the burden associated with their comorbidity in different contexts, with future research directions proposed.

**Systematic review registration:**

This protocol is registered in Open Science Framework Registries (https://osf.io/mxbr2/).

**Supplementary Information:**

The online version contains supplementary material available at 10.1186/s13643-021-01721-6.

## Introduction

Separately, mental and musculoskeletal disorders (MSDs) are prevalent across the life course and are the leading contributors to disability worldwide [1, 2]. In 2010, mental disorders [1, 3] were the leading cause of years lived with disability (YLDs), with MSDs being the second [4]. By 2017, major depressive disorder (MDD) was identified as the main driver for mental disorder-related disability, with low back pain the leading cause of the musculoskeletal-related disability, but the order of these two main disease classes remained the same in respect to YLDs [2]. Combined, mental disorders and MSDs are now estimated to account for over 30% of the global YLDs [5, 6]. Little improvement in population health is reported in relation to these disorders—and the associated burden is expected to rise, given increasing population growth and ageing [1, 2].

Recently, a review of the literature reported associations *between* mental disorders and MSDs among middle-aged and older adults showing that those with depressive and anxiety disorders and poor subjective mental health had compromised bone health and increased risk of osteoporotic fractures, respectively [5]. Furthermore, certain medications used in the treatment of mental disorders have also been shown to independently affect bone [7]. Elsewhere, there is a suggestion that the risk of falling, and associated fracture, may be exacerbated by the presence of depression [7, 8] and other types of mental ill health [9, 10]. However, personality disorder (PD), an often-severe form of mental ill health, with far-reaching public health implications including comorbidity with other physical health conditions [11], has not been thoroughly explored in relation to MSDs. This is despite burgeoning research showing PD co-occurs with sleep disorders, headache and pain disorders, obesity, and other chronic health conditions [11]. People with PD symptomology also perceive their overall health to be worse than people without [12] and report difficulties with general health care compliance [13]. Separately, PD is also associated with high direct medical costs, and high costs, due to productivity loss [14]. However, little is known regarding the burden associated with PD and MSD comorbidity specifically.

By definition, PD is marked by patterns of distressing behaviours, ways of thinking, emotionality, and inner experiences that differ significantly from those in one’s usual social/cultural context—causing significant impairment in important areas of life and distress [15]. PD usually emerges earlier in the lifespan and can be relatively more enduring, inflexible, and disabling than other forms of mental ill health [15]. Traditionally, specific PDs include paranoid, schizoid, schizotypal, antisocial, borderline, histrionic, narcissistic, avoidant, dependent, and obsessive–compulsive PDs [15]. However, there is variation between the two main classification systems (i.e. International Classification of Diseases [ICD] and Diagnostic and Statistical Manual of Mental Disorders [DSM]), in terms of definitions and approaches to diagnosis—with longstanding and unresolved commentary in the literature concerning these issues [16, 17]. Still, their clinical significance is not contested, and exploring their relationship to MSDs and the burden associated with their comorbidity is warranted, given the known links between PD and other adverse physical health outcomes.

A preliminary search revealed no prior review on the proposed topic. One systematic review, however, was found, which reported that people with serious mental illness including schizophrenia, bipolar disorders, depression, and depression *with* borderline PD had lowered bone mineral density [18]. However, the authors chiefly examined osteoporosis or fractures as outcomes of interests, and other types of burdensome MSDs were not explored. Also, given that PD was not examined as a risk factor per se, studies with a focus on PD in relation to MSDs may have gone undetected. The proposed scoping review will be a valuable contribution to the literature, as it will broaden the currently narrow research focus on common mental disorders and physical comorbidities—and stimulate further research on this topic.

Therefore, we propose to undertake a scoping review to explore the existing clinical- and population-based literature, the comorbidity of PD and MSDs among adults ≥ 18 years, and the potential burden associated with their comorbidity; identify knowledge gaps on this topic; and propose recommendations for future research.

## Methods

This protocol is guided by Arksey and O’Malley’s five-stage methodological framework [19]—and the extensions to this original framework developed by the Joanna Briggs Institute (JBI) [20, 21]. This protocol is registered in Open Science Framework Registries (https://osf.io/mxbr2/) and complies, where relevant, to the Preferred reporting items for systematic review and meta-analysis protocols (PRISMA-P) 2015 checklist (see Additional file).

This protocol is structured as follows [19]:

- Stage 1: Identifying the research question
- Stage 2: Identifying relevant studies
- Stage 3: Study selection
- Stage 4: Charting the data
- Stage 5: Collating, summarising, and reporting the results

The final scoping review will adhere to and present the PRISMA extension for Scoping Reviews checklist as supplementary material [22]. Each stage is described in more detail in the following sections.

### Stage 1: Identifying the research question

Initially, the development of the research questions was guided by an existing review [5]. The purpose of the previously published review was to examine anxiety and depressive disorders and subjective mental health—but not PD—among middle-aged and older adults in relation to MSDs [5]. Building on the previous review, the authors’ prior knowledge of associations between PD and adverse physical health outcomes and given that mental disorders and MSDs are leading causes of YLDs, the authors identified PD as the specific mental disorder of interest for this scoping review. A decision was made by the authors to examine the full-adult age range, given that PD may be a risk to musculoskeletal health earlier and across the lifespan. From here, the following indicative research questions were developed:

- What is known from the existing clinical- and population-based literature regarding associations between PD and MSDs?
- What is known from the existing literature regarding disease burden associated with the comorbidity of PD and MSDs?
- What are the knowledge gaps in relation to this topic?
- What recommendations for future research can be made?

An iterative approach will be taken to refining the research questions. For example, the indicative research questions may be further refined, or added to, as the authors develop in-depth knowledge of the topic [5].

### Stage 2: Identifying relevant studies

This section describes the process for identifying studies for consideration for the proposed scoping review. First, the authors developed key inclusion criteria using the “Population–Concept–Context (PCC)” framework recommended by JBI for scoping reviews, which is an adaption of the population, intervention, comparator, and outcome framework—and commonly used to develop systematic search strategies [20].

A summary of the PCC inclusion criteria is presented in Table 1.

**Table 1:** Summary of the PCC inclusion criteria

Inclusion criteria	
**P—population**	
Studies will be considered if they examine:	
• Adults ≥ 18 years who have any PD or PD features/traits/dysfunction/pathology according to current classifications systems, as identified by a relevant health professional/otherwise identified	
**C—concept**	
Studies will be considered if they examine adults with PD in relation to:	
• MSDs according to the broad World Health Organization (WHO) definition including falls, as diagnosed by a physician/otherwise identified	
• Disease burden associated with PD and MSD comorbidity	
**C—context**	
Worldwide studies with observational study designs from clinical or population-based contexts.	

#### Inclusion criteria

Studies will be considered if they examine adults who:

- Are ≥ 18 years (young people whose age overlaps with adulthood [i.e. 15–24 years] may be considered)
- Have any PD or PD features/traits/dysfunction/pathology according to the two main classifications systems (e.g. ICD or DSM), which has been identified by a relevant health professional (i.e. a physician, psychiatrist, psychologist, or allied health care professional), medical records/registries, diagnostic interviews performed by trained researchers, and/or self-administered questionnaires/reported

Studies will be considered if they examine the population of interest in relation to MSDs, which have been diagnosed by a physician or otherwise identified (i.e. medical records/registries, and/or self-administered questionnaires/reported).

We define MSDs according to the broad World Health Organization (WHO) definition including conditions that affect [23]:

- Joints (e.g. osteoarthritis, rheumatoid arthritis, psoriatic arthritis, gout, ankylosing spondylitis)
- Bones (e.g. osteoporosis, osteopenia, fragility fractures, traumatic fractures)
- Muscles (e.g. sarcopenia)
- Spine (e.g. low back/neck pain)
- Body areas/systems (e.g. regional/widespread pain disorders such as fibromyalgia/inflammatory diseases with musculoskeletal manifestations)

Falls will also be included in our broad definition, given their association with other types of mental disorders, and injurious consequences, respectively [8, 9, 24]. Studies will also be considered if they examine the burden of PD and MSD comorbidity, which may include:

- Comorbidity or morbidity
- Patient-reported outcomes (e.g. pain, subjective wellbeing, symptomatology)
- Clinician-reported outcomes (e.g. remission status)
- Work-related outcomes (e.g. work disability status)
- Number of hospital admissions/length of stay
- Mortality
- Financial costs (e.g. direct or indirect health care costs as described in the available literature)
- Other indicators such as disability-adjusted life years (DALY), quality-adjusted life years (QALYs), or YLDs

Finally, the proposed scoping review will consider studies with observational study designs or reviews citing observational studies from the following contexts:

- Worldwide
- Clinical settings
- Population-based settings

There will be no date restrictions applied. However, only studies published in English in peer-reviewed journal articles will be considered. Grey literature may be considered if shown to address the research questions. Finally, studies examining PD in relation to *intentional injuries* are considered out of scope.

#### Search strategy

A comprehensive search strategy will be developed to identify relevant articles, which will be underpinned by our key inclusion criteria—and the process recommended by the JBI [20].

First, we conducted a preliminary search for articles on the proposed topic in Google Scholar, PROSPERO, PubMed, the Cochrane Database of Systematic Reviews, JBI Evidence Synthesis, and Open Registries, which revealed no prior reviews.

As a starting point, and to derive a list of potentially relevant search terms, the indicative search strategy (see Table 2) was informed by an existing review on the topic of depressive and anxiety disorders, and subjective wellbeing in relation to MSDs [5], as well as key papers known to the authors on the scoping review topic [25–31]. This list was expanded using a combination of Medical Subject Headings (MeSH) and keywords, which were relevant to the PCC inclusion criteria. An academic librarian/information specialist reviewed the indicative search strategy and will be consulted to evaluate the final strategy as guided by the Peer Review of Electronic Search Strategies checklist [32]. Further refinement may involve using additional MeSH, keywords, truncations (stemming), and/or wildcards, with Boolean operators (OR, AND), where appropriate.

**Table 2:** Indicative search strategy for MEDLINE Complete via EBSCOhost

Search line	Index terms/keyword/combinations
**S1**	(MH “Personality Disorders+”)
**S2**	(AB “personality disorder*”)
**S3**	(TI “personality disorder*”)
**S4**	((TI personality OR TI borderline) AND (TI disorder* OR TI dysfunction* OR TI pathology OR TI feature* OR TI trait* OR TI symptom*))
**S5**	(MH “Musculoskeletal Diseases+”)
**S6**	(AB musculoskeletal)
**S7**	(TI musculoskeletal)
**S8**	(MH “Bone Density”)
**S9**	(AB bone*)
**S10**	(TI bone*)
**S11**	(MH “Fractures, Bone+”)
**S12**	(AB fracture*)
**S13**	(TI fracture*)
**S14**	(MH “Accidental Falls”)
**S15**	(AB fall*)
**S16**	(TI fall*)
**S17**	((TI physical OR TI medical OR TI chronic) AND (TI illness* OR TI disease* OR TI condition* OR TI comorbidity OR TI problem))
**S18**	((TI musculoskeletal* OR TI bone* OR TI fall* OR TI fracture*) AND ((TI disease AND TI burden)) OR (TI morbidity OR TI multimorbitity OR TI mortality OR TI disability* OR TI cost*))
**S19**	S1 OR S2 OR S3 OR S4
**S20**	S5 OR S6 OR S7 OR S8 OR S9 OR S10 OR S11 OR S12 OR S13 OR S15 OR S16 OR S17 OR S18
**S21**	S19 AND S20

Search modes = Boolean/Phrase. Search options = Expanders; apply equivalent subjects. Search fields = search in abstract field (AB); search in MeSH/Index Term field (MH); search title field (TI)

The search strategy will be translated for each specific database searched including MEDLINE, CINAHL, and PsycINFO databases via the EBSCOhost online research platform. Grey literature will be searched using an adapted query in Google. Further sources of evidence may be found by “snowballing”, including searching referencing lists of identified studies, citation tracking, and/or through existing networks. The ensuing scoping review will provide complete details concerning the final search strategy and results, including the date of the searches and the date last executed, as well as any search limitations/filters applied.

### Stage 3: Study selection

One author will perform and consolidate the results from the separate searches and remove duplicate records. The records will be managed using Covidence [33] and a reference management software such as Mendeley. Two reviewers will screen titles and/or abstracts of the articles retrieved and exclude those that are not relevant according to the PCC inclusion criteria. Full-text articles will be retrieved for records considered relevant and assessed independently by the same reviewers to ensure consistent application of the PCC inclusion criteria. Any potential disagreements concerning eligibility will be discussed between the two authors, and the supervising author will provide the final decision to reach consensus. The final inclusion of full-text articles will be cross-checked against the PCC inclusion criteria and confirmed by the supervising author.

### Stage 4: Charting the data

To address the research questions, a charting form (see Table 3), which will be adapted from the *JBI template source of evidence details, characteristics, and results extraction instrument* [20], will be used to extract relevant information from identified observational studies or grey literature. For review studies, the authors may source the data from the original article(s), if relevant. For the purposes of developing a descriptive summary of the findings, the indicative charting form will capture basic citation details, PCC information, study approach/methodology, key results, summary of key findings, study limitations, and identified knowledge gaps (either reported in the article or identified by the reviewers). A code will be applied for missing or not applicable information.

**Table 3:** Indicative charting form

Citation details (e.g. author; country; year)	Setting/context (e.g. clinical- or population-based)	Population characteristics (e.g. age, sex, psychiatric comorbidity)	Methods (e.g. study design/method)	Assessment of PD/psychiatric comorbidity (e.g. diagnostic interview; DSM-5)	Assessment of MSDs (e.g. osteoporosis; identified by physician)	Indicator of disease burden (e.g. DALYs)	Key results (e.g. ORs/IRR according to each relevant concept/outcome; adjusted/unadjusted models noted)	Summary of key findings (e.g. description of the key findings)	Study limitations (e.g. reported by authors or otherwise identified)	Identified knowledge gaps (e.g. reported by authors or otherwise identified)	Recommendation for future research (e.g. reported by authors or otherwise identified)
.	.	.	.	.	.	.	.	.	.	.	.

Two reviewers will independently pilot the charting form with a sample of studies to ensure that it is appropriate to address the research questions. For example, the reviewers will independently extract relevant information from five articles and hold a consensus meeting with the supervisor author to discuss the results.

The same two reviewers will be involved in the charting process; one will extract the information and the other will perform a validation task. Any potential errors detected during the validation step will be corrected. Due to time constraints, authors of published studies will not be contacted for data requests or clarifications. Emerging themes in the literature and/or the need to modify the charting form will also be discussed with and confirmed by the whole group via fortnightly videoconferencing and/or emails throughout the conduct of the review. Any modification to the charting form or process will be detailed in the full scoping review. All authors will contribute to the interpretation of the information extracted.

### Stage 5: Collating, summarising, and reporting the results

The results will be presented in figures, tables, and text. The results of the search strategy and selection process will be presented in a flow diagram and summarised in text. In addition, the characteristics of all studies will be described in text and presented in a table. Furthermore, the main results will be presented in a descriptive synthesis and according to each research question. The descriptive synthesis will also highlight study limitations, knowledge gaps, and areas that may warrant further research relevant to the scoping review topic.

## Discussion

The proposed scoping review will extend existing reviews by employing a robust search strategy to explore, uncover, and bring together in one review what is known regarding the comorbidity of PD and MSDs from published and unpublished evidence sources in both population-based and clinical settings. Uncovering and synthesising the available evidence on this topic may prompt future research including systematic reviews and meta-analyses. It may also reveal literature that describes or postulates the underlying connection between PD and MSDs, and/or the burden associated with their comorbidity. These insights may lead to an improved understanding of the experiences of people with these comorbidities and treatment targets.

In terms of dissemination, the ensuing scoping review will be submitted for publication in a scientific, peer-reviewed journal. The findings from the proposed scoping review may be presented at relevant conferences and used to inform the development of future research studies.

In terms of potential limitations, the nature of the scoping review is exploratory, and the reviewed studies will likely vary widely in terms of their methodology—including definitions of PD, all of which preclude a systematic review or meta-analyses [21, 34]. To overcome these restraints, a scoping review methodology will be the most appropriate methodology to address the research objectives, given the knowledge base on this topic is still emerging and not well understood. Consistent with published guidance [35], critical appraisal will not be performed on eligible studies. However, a summary of the strengths and limitations of reviewed studies will be reported in the discussion. Ethics approval is not required for this scoping review.

## Conclusion

The proposed scoping review will valuably contribute to the literature as it will be the first review to explore and provide a descriptive synthesis of what is known regarding the comorbidity of PD and MSDs among adults in different contexts—while offering future research directions.

## Supplementary Information

**Additional file 1.** PRISMA-P 2015 Checklist.

## Data Availability

Not applicable for this protocol.

## Abbreviations

DSM: Diagnostic and Statistical Manual of Mental Disorders
JBI: Joanna Briggs Institute
ICD: International Classification of Diseases
MSDs: Musculoskeletal disorders
PD: Personality disorder
PCC framework: Population–Concept–Context framework

## References

[1] Whiteford HA, Degenhardt L, Rehm J, Baxter AJ, Ferrari AJ, Erskine HE, Charlson FJ, Norman RE, Flaxman AD, Johns N, Burstein R, Murray CJL, Vos T (2013). Global burden of disease attributable to mental and substance use disorders: findings from the Global Burden of Disease Study 2010. Lancet.

[2] Vos T, Abajobir AA, Abbafati C, Abbas KM, Abate KH, Abd-Allah F (2017). Global, regional, and national incidence, prevalence, and years lived with disability for 328 diseases and injuries for 195 countries, 1990-2016: a systematic analysis for the Global Burden of Disease Study 2016. Lancet.

[3] Lim SS, Vos T, Flaxman AD, Danaei G, Shibuya K, Adair-Rohani H, AlMazroa MA, Amann M, Anderson HR, Andrews KG, Aryee M, Atkinson C, Bacchus LJ, Bahalim AN, Balakrishnan K, Balmes J, Barker-Collo S, Baxter A, Bell ML, Blore JD, Blyth F, Bonner C, Borges G, Bourne R, Boussinesq M, Brauer M, Brooks P, Bruce NG, Brunekreef B, Bryan-Hancock C, Bucello C, Buchbinder R, Bull F, Burnett RT, Byers TE, Calabria B, Carapetis J, Carnahan E, Chafe Z, Charlson F, Chen H, Chen JS, Cheng ATA, Child JC, Cohen A, Colson KE, Cowie BC, Darby S, Darling S, Davis A, Degenhardt L, Dentener F, Des Jarlais DC, Devries K, Dherani M, Ding EL, Dorsey ER, Driscoll T, Edmond K, Ali SE, Engell RE, Erwin PJ, Fahimi S, Falder G, Farzadfar F, Ferrari A, Finucane MM, Flaxman S, Fowkes FGR, Freedman G, Freeman MK, Gakidou E, Ghosh S, Giovannucci E, Gmel G, Graham K, Grainger R, Grant B, Gunnell D, Gutierrez HR, Hall W, Hoek HW, Hogan A, Hosgood HD, Hoy D, Hu H, Hubbell BJ, Hutchings SJ, Ibeanusi SE, Jacklyn GL, Jasrasaria R, Jonas JB, Kan H, Kanis JA, Kassebaum N, Kawakami N, Khang YH, Khatibzadeh S, Khoo JP, Kok C, Laden F, Lalloo R, Lan Q, Lathlean T, Leasher JL, Leigh J, Li Y, Lin JK, Lipshultz SE, London S, Lozano R, Lu Y, Mak J, Malekzadeh R, Mallinger L, Marcenes W, March L, Marks R, Martin R, McGale P, McGrath J, Mehta S, Memish ZA, Mensah GA, Merriman TR, Micha R, Michaud C, Mishra V, Hanafiah KM, Mokdad AA, Morawska L, Mozaffarian D, Murphy T, Naghavi M, Neal B, Nelson PK, Nolla JM, Norman R, Olives C, Omer SB, Orchard J, Osborne R, Ostro B, Page A, Pandey KD, Parry CDH, Passmore E, Patra J, Pearce N, Pelizzari PM, Petzold M, Phillips MR, Pope D, Pope CA, Powles J, Rao M, Razavi H, Rehfuess EA, Rehm JT, Ritz B, Rivara FP, Roberts T, Robinson C, Rodriguez-Portales JA, Romieu I, Room R, Rosenfeld LC, Roy A, Rushton L, Salomon JA, Sampson U, Sanchez-Riera L, Sanman E, Sapkota A, Seedat S, Shi P, Shield K, Shivakoti R, Singh GM, Sleet DA, Smith E, Smith KR, Stapelberg NJC, Steenland K, Stöckl H, Stovner LJ, Straif K, Straney L, Thurston GD, Tran JH, van Dingenen R, van Donkelaar A, Veerman JL, Vijayakumar L, Weintraub R, Weissman MM, White RA, Whiteford H, Wiersma ST, Wilkinson JD, Williams HC, Williams W, Wilson N, Woolf AD, Yip P, Zielinski JM, Lopez AD, Murray CJL, Ezzati M (2012). A comparative risk assessment of burden of disease and injury attributable to 67 risk factors and risk factor clusters in 21 regions, 1990-2010: a systematic analysis for the Global Burden of Disease Study 2010. Lancet.

[4] Vos T (2012). Years lived with disability (YLDs) for 1160 sequelae of 289 diseases and injuries 1990-2010: a systematic analysis for the Global Burden of Disease Study 2010. Lancet.

[5] Heikkinen J, Honkanen R, Williams L, Leung J, Rauma P, Quirk S, Koivumaa-Honkanen H (2019). Depressive disorders, anxiety disorders and subjective mental health in common musculoskeletal diseases: a review. Maturitas.

[6] Institute for Health Metrics and Evaluation (2019). Global Burden of Disease (GBD) results tool.

[7] Fernandes BS, Hodge JM, Pasco JA, Berk M, Williams LJ (2016). Effects of depression and serotonergic antidepressants on bone: mechanisms and implications for the treatment of depression. Drugs Aging.

[8] Stuart AL, Pasco JA, Jacka FN, Berk M, Williams LJ (2018). Falls and depression in men: a population-based study. Am J Mens Health.

[9] Bunn F, Dickinson A, Simpson C, Narayanan V, Humphrey D, Griffiths C, et al. Preventing falls among older people with mental health problems: a systematic review. BMC Nurs. 2014;13(1). 10.1186/1472-6955-13-4. 10.1186/1472-6955-13-4 PMC3942767 24552165

[10] Williams LJ, Pasco JA, Stuart AL, Jacka FN, Brennan SL, Dobbins AG (2015). Psychiatric disorders, psychotropic medication use and falls among women: an observational study. BMC Psychiatry.

[11] Dixon-Gordon KL, Whalen DJ, Layden BK, Chapman AL (2015). A systematic review of personality disorders and health outcomes. Can Psychol.

[12] Fok M, Hotopf M, Stewart R, Hatch S, Hayes R, Moran P (2014). Personality disorder and self-rated health: a population-based cross-sectional survey. J Pers Disord.

[13] Sansone RA, Bohinc RJ, Wiederman MW (2015). Borderline personality symptomatology and compliance with general health care among internal medicine outpatients. Int J Psychiatry Clin Pract.

[14] Soeteman DI, Hakkaart-van Roijen L, Verheul R, Busschbach JJ (2008). The economic burden of personality disorders in mental health care. J Clin Psychiatry.

[15] American Psychiatric Association. Diagnostic and statistical manual of mental disorders (5th ed.). 2013. 10.1176/appi.books.9780890425596.

[16] Tyrer P, Mulder R, Kim Y-R, Crawford MJ (2019). The development of the ICD-11 classification of personality disorders: an amalgam of science, pragmatism, and politics. Annu Rev.

[17] Herpertz SC, Huprich SK, Bohus M, Chanen A, Goodman M, Mehlum L, Moran P, Newton-Howes G, Scott L, Sharp C (2017). The challenge of transforming the diagnostic system of personality disorders. J Pers Disord..

[18] Xie H, Loh S, Shan CP, Wang J, Peng Y, Fen LQ (2012). Osteoporosis in adults with mental illnesses: a systematic review. JBI Libr Syst Rev.

[19] Arksey H, O’Malley L (2005). Scoping studies: towards a methodological framework. Int J Soc Res Methodol Theory Pract.

[20] Joanna Briggs Institute. The Joanna Briggs Institute Reviewer’s manual. Chapter 11: scoping reviews. https://wiki.joannabriggs.org/display/MANUAL/Chapter+11%3A+Scoping+reviews. Accessed 3 Apr 2020.

[21] Munn Z, Peters MDJJ, Stern C, Tufanaru C, McArthur A, Aromataris E (2018). Systematic review or scoping review? Guidance for authors when choosing between a systematic or scoping review approach. BMC Med Res Methodol.

[22] Tricco AC, Lillie E, Zarin W, O’Brien KK, Colquhoun H, Levac D (2018). PRISMA extension for scoping reviews (PRISMA-ScR): checklist and explanation. Ann Intern Med.

[23] World Health Organization. Musculoskeletal conditions. https://www.who.int/news-room/fact-sheets/detail/musculoskeletal-conditions. Accessed 3 Apr 2020.

[24] Biderman A, Cwikel J, Fried AV, Galinsky D (2002). Depression and falls among community dwelling elderly people: a search for common risk factors. J Epidemiol Community Health.

[25] Quirk SE, El-Gabalawy R, Brennan SL, Bolton JM, Sareen J, Berk M (2015). Personality disorders and physical comorbidities in adults from the United States: data from the National Epidemiologic Survey on Alcohol and Related Conditions. Soc Psychiatry Psychiatr Epidemiol.

[26] El-Gabalawy R, Katz LY, Sareen J (2010). Comorbidity and associated severity of borderline personality disorder and physical health conditions in a nationally representative sample. Psychosom Med.

[27] Kahl KG, Rudolf S, Stoeckelhuber BM, Dibbelt L, Gehl H-BB, Hohagen F (2005). Bone mineral density, markers of bone turnover, and cytokines in young women with borderline personality disorder with and without comorbid major depressive disorder. Am J Psychiatry.

[28] Kahl KG, Greggersen W, Rudolf S, Stoeckelhuber BM, Bergmann-Koester CU, Dibbelt L, Schweiger U (2006). Bone mineral density, bone turnover, and osteoprotegerin in depressed women with and without borderline personality disorder. Psychosom Med.

[29] Keuroghlian AS, Frankenburg FR, Zanarini MC (2013). The relationship of chronic medical illnesses, poor health-related lifestyle choices, and health care utilization to recovery status in borderline patients over a decade of prospective follow-up. J Psychiatr Res.

[30] Powers AD, Oltmanns TF (2012). Personality disorders and physical health: a longitudinal examination of physical functioning, healthcare utilization, and health-related behaviors in middle-aged adults. J Pers Disord.

[31] Quirk SE, Stuart AL, Brennan-Olsen SL, Pasco JA, Berk M, Chanen AM (2016). Physical health comorbidities in women with personality disorder: data from the Geelong Osteoporosis Study. Eur Psychiatry.

[32] McGowan J, Sampson M, Salzwedel DM, Cogo E, Foerster V, Lefebvre C (2016). PRESS peer review of electronic search strategies: 2015 guideline statement.

[33] Covidence. Covidence systematic review software, Veritas Health Innovation, Melbourne, Australia. https://www.covidence.org/.

[34] Peters MDJ, Godfrey CM, Khalil H, McInerney P, Parker D, Soares CB (2015). Guidance for conducting systematic scoping reviews. Int J Evid Based Healthc.

[35] Peters MDJ, Marnie C, Tricco AC, Pollock D, Munn Z, Alexander L (2020). Updated methodological guidance for the conduct of scoping reviews. JBI Evid Synth.

